# The opportunities and challenges of using *Drosophila* to model human cardiac diseases

**DOI:** 10.3389/fphys.2023.1182610

**Published:** 2023-04-12

**Authors:** Yunpo Zhao, Joyce van de Leemput, Zhe Han

**Affiliations:** ^1^ Center for Precision Disease Modeling, Department of Medicine, University of Maryland School of Medicine, Baltimore, MD, United States; ^2^ Division of Endocrinology, Diabetes and Nutrition, Department of Medicine, University of Maryland School of Medicine, Baltimore, MD, United States

**Keywords:** congenital heart disease, hypertrophic cardiomyopathy, cardiac arrhythmia, *Drosophila*, cardiac conduction, cardiac physiology

## Abstract

The *Drosophila* heart tube seems simple, yet it has notable anatomic complexity and contains highly specialized structures. In fact, the development of the fly heart tube much resembles that of the earliest stages of mammalian heart development, and the molecular-genetic mechanisms driving these processes are highly conserved between flies and humans. Combined with the fly’s unmatched genetic tools and a wide variety of techniques to assay both structure and function in the living fly heart, these attributes have made *Drosophila* a valuable model system for studying human heart development and disease. This perspective focuses on the functional and physiological similarities between fly and human hearts. Further, it discusses current limitations in using the fly, as well as promising prospects to expand the capabilities of *Drosophila* as a research model for studying human cardiac diseases.

## Introduction

**FIGURE 1:**
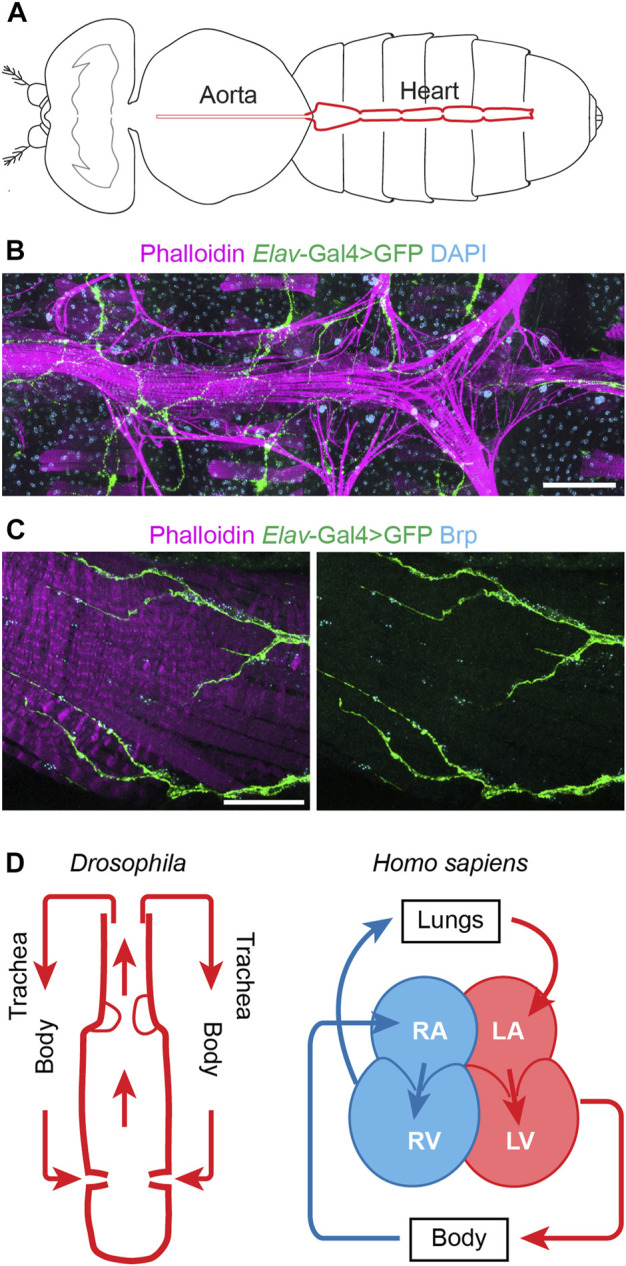
The *Drosophila* and human hearts. **(A)** Schematic illustration of an adult fly (dorsal view). The aorta and the heart are shown in red. **(B)** Representative image of a *Drosophila* adult heart (ventral view). Phalloidin stains filament actin in red. The *elav*-Gal4>GFP (*elav, embryonic legal abnormal vision*; GFP, green fluorescent protein) labels cardiac neuron fiber in green. DAPI stains DNA in blue. The scale bar represents 100 μm. Image was obtained using a ZEISS Apotome fluorescence microscope using a 20× objective and ZEISS Zen (blue edition) acquisition software. **(C)** Representative image of the posterior *Drosophila* adult heart (ventral view). Phalloidin stains filament actin in red. The *elav*-Gal4>GFP (*elav, embryonic legal abnormal vision*; GFP, green fluorescent protein) labels cardiac neuronal axons in green. Brp, Bruchpilot, is in blue and labels the neuromuscular junctions. The scale bar represents 20 μm. Images were obtained using a ZEISS LSM900 confocal microscope using a 63× objective and ZEISS Zen (blue edition) acquisition software. **(D)** Schematic representations of the fly (*Drosophila*) and human (*Homo sapiens*) hearts. Arrows indicate the direction of flow for the fly hemolymph and human blood (blue, oxygen poor; red, oxygen rich blood). Structure inside the fly heart depicts the aortic valve, and flow enters the heart at the inflow tracts with ostia (valves). Human heart chambers: RA, right atrium; RV, right ventricle; LA, left atrium; LV, left ventricle.

At first sight flies, and by extension their physiology, seem far removed from humans, yet the fly’s simple heart tube harbors more complexity than initially assumed. The *Drosophila* heart has distinct morphological features, including an anterior aorta structure that is separated from the posterior heart chamber by an aortic valve to ensure posterior to anterior flow, and it has inflow tracts named ostia (Bodmer, 1995; Rotstein and Paululat, 2016) (Figure 1). One notable difference is that fly has an open circulatory system, *i.e.*, all hemolymph is oxygenated (akin mammalian arterial blood) and flows via channel-like trajectories formed by the internal organs and by fibromuscular septa or diaphragms (Hillyer and Pass, 2020), with the whole body acting as trachea (Figure 1). Despite this difference, the earliest stages of heart development are extremely well conserved from flies to humans. These cover the migration of the bilateral rows of cardiac progenitor cells towards the midline to their fusion to form the heart tube (Bodmer, 1995; Ahmad, 2017). These early similarities go beyond structure to include molecular genetics (Olson, 2006; Souidi and Jagla, 2021). In fact, the first gene known to control heart development, *tinman* (*tin*), was discovered in flies (Azpiazu and Frasch, 1993; Bodmer, 1993). This then led to the identification of its homolog, *Nkx2.5*, as a key transcription factor for mammalian heart development (Komuro and Izumo, 1993; Lyons et al., 1995; Tanaka et al., 1999). Many of the major genetic pathways in human heart development, like *WNT*, *TGFβ*, and *FGF*, as well as key transcription factors, like *NKX2.5*, *QRSL1 (a.k.a. GATA)*, *TBX* (*T-box*), *MEF2*, and the *HAND* family, are evolutionarily conserved and have homologs in flies, zebrafish, and mice (Bodmer, 1995; Olson, 2006; Cui et al., 2018). For example, *Wnt4* is required for the development of the ostia in the fly heart (Chen et al., 2016); and Wnt signaling is a key regulator of mammalian heart development, during which Wnt mediates cardiac specification, proliferation, and patterning (Gessert and Kühl, 2010; Li D. et al., 2022). The shared developmental genetic, molecular, cellular, and functional mechanisms culminate in shared physiology. Therefore, despite the simpler heart structure in fly and the evolutionary distance between flies and humans, the fly heart’s structural and functional similarities to the human heart during early development combined with its genetic tools and resources have made *Drosophila* a valuable model system to study human cardiac diseases (to highlight a few: Qian et al., 2008; Ma et al., 2010; Neely et al., 2010; den Hoed et al., 2013; Haack et al., 2013; Zhu et al., 2017a; Iuso et al., 2018; Kronert et al., 2018; Palandri et al., 2018; Ekure et al., 2021) (Table 1).

**TABLE 1:** *Drosophila* models of human cardiac diseases.

Cardiac pathology	Patient physiology	Fly physiology	References^1^
Congenital heart diseases	Various structural and functional cardiac defects; Marked by early onset (*i.e.*, cardiac defects present at birth)	Developmental lethality; Structural defects, include absent heart structure, reduced cardiac myofibers, and increased Pericardin deposition; Functional defects include reduced/abolished contractility	Qian et al. (2008), Zhu et al. (2017a), Schroeder et al. (2019), Ekure et al. (2021)
Cardiomyopathies	Various structural defects, include increased ventricular size, thickening of the ventricles (*i.e.*, hypertrophy), and stiffening of the ventricular walls; Various cardiac functional defects, include systolic and diastolic dysfunction, arrhythmia, heart failure; May have congenital or pediatric onset	Developmental lethality; Structural defects, include increased chamber size, and cardiac hypertrophy; Cardiac functional defects, include reduced contraction, arrhythmia, increased heart rate, reduced systolic function, and fractional shortening	Ma et al. (2010), Neely et al. (2010), Yu et al., 2010, Yu et al. (2013), en Hoed et al. (2013), Haack et al. (2013), Iuso et al. (2018), Kronert et al. (2018), Palandri et al. (2018), Manivannan et al. (2020), Migunova et al. (2021)
Metabolic-associated heart diseases	Obesity-associated cardiomyopathy marked by altered metabolism: Structural defects include ventricular hypertrophy and remodeling; Functional defects include diastolic and systolic dysfunction, abnormal conduction, arrhythmia, and heart failure	High-fat diet obesity model marked by altered metabolism: Structural defects include disorganized myofibrils; Cardiac functional defects, include reduced contractility, conduction blocks, and dysfunctional ostia; Phenotype persisted for two subsequent generations	Birse et al. (2010), Guida et al. (2019)
	Diabetes-associated dilated cardiomyopathy marked by hyperglycemia, hypertrehalosemia, peripheral resistance to exogenous insulin, and accumulation of triglyceride; Structural defects include increased heart ventricular size, and cardiac fibrosis; Cardiac functional defects include altered systole and diastole, arrhythmia	High-sugar diet diabetes model marked by hyperglycemia, hypertrehalosemia, peripheral resistance to exogenous insulin, and accumulation of triglyceride; Shortened lifespan; Structural defects include increased Pericardin deposition; Cardiac functional defects, include increased diastole and systole (without fractional shortening), and arrhythmia; Maternal inheritance	Na et al. (2013), Basu et al. (2017)
Aging-associated cardiac decline	Cardiac functional decline includes arrhythmia, and decreased diastolic function	Cardiac functional decline includes reduced resting heart rate, arrhythmia, and decreased cardiac output, as well as increased risk of stress-induced heart failure	Paternostro et al. (2001), Wessells et al. (2004), Wessells et al. (2009), Luong et al. (2006), Ocorr et al. (2007), Taghli-Lamallem et al. (2008), Cannon et al. (2017), Klassen et al. (2017)

^1^ The listed references are a selection of the available literature. Unfortunately, it is outside the scope of this perspective to discuss all available studies.

## *Drosophila* models of congenital heart disease (CHD)

CHD affects over 1% of all live births, making it the most frequent type of birth defect (Pierpont et al., 2018). Although the contribution of genetics to CHD has been well-established, identifying the causal genetic mutations for individual CHD patients has proven difficult. Tremendous effort has been invested to identify CHD-linked causal variants. For example, the Pediatric Cardiac Genomic Consortium (PCGC) (Zaidi et al., 2013; Homsy et al., 2015; Jin et al., 2017) incorporates CHD-related findings from many studies including Kids First, Center for Mendelian Genomics (CMG), Undiagnosed Disease Network (UDN) (Posey et al., 2019), and Deciphering Developmental Disorders (DDD) (Sifrim et al., 2016; Verheije et al., 2019). These large-scale genomic sequencing projects, together with studies led by cardiologists and medical geneticists, have identified thousands of novel candidate genes and variants in patients with CHD.

In the absence of independent patient families that each carry the same genetic variant, functional validation is essential to establish causality. Screening such a large number of candidate CHD genes is not feasible using conventional mammalian research models, which are too time-consuming and costly for these purposes. *Drosophila* could bridge this gap because most disease-causing genes have homologs in fly (Ugur et al., 2016). For example, the highly conserved *NKX2.5* (*tinman* in fly) which plays a crucial role in heart development is also a hotspot for genetic variants that have been linked to CHD (McElhinney et al., 2004; Pashmforoush et al., 2004). In addition, the fly has an unprecedented arsenal of genetic tools that enable precise genetic manipulation to target specific developmental time points, or specific tissues and even cell-types within those tissues (Hales et al., 2015; Zhao et al., 2021). Over the years many techniques to assay heart structure and function in the fly have been developed; these have consistently shown the physiological similarities of *Drosophila* and human heart development and function, as well as dysfunction when studying disease-associated genes and variants (Wolf and Rockman, 2008; Manivannan et al., 2020). Altogether, these features make the fly a versatile model system, capable of rapid cost-effective screens of hundreds of candidate genetic variants for CHD. One such study used an RNAi-based functional screen of 134 genes associated with CHD, of which over 70 genes were shown to be involved in *Drosophila* heart development thus supporting their causality (Zhu et al., 2017a). One of the hits was *WD repeat domain 5* (*WDR5*); silencing its homolog *Wds* in the fly heart caused complete developmental lethality and abnormal cardiac morphology in late larvae, including reduced cardiac myofibers and increased Pericardin deposition (Zhu et al., 2017a). Notably, overexpressing wildtype human *WDR5* restored the cardiac phenotype in flies with heart-specific deficiency for *Wds*, whereas human *WDR5* carrying a patient variant could not (Zhu et al., 2017a). These findings demonstrate the physiological (gene-structure) homology between human and fly key cardiac genes. Another study identified 19 deleted *de novo* copy number variants (CNVs) covering hitherto not associated candidate disease genes in a cohort of 167 patients with CHD (Schroeder et al., 2019). These were then tested using parallel screens in human induced pluripotent stem cell (iPSC)-derived multipotent cardiac progenitor cells and a *Drosophila in vivo* heart model. Flies with heart-specific deficiency for candidate genes showed phenotypes ranging from a completely absent heart to structural and functional defects that included reduced or abolished contractility (Schroeder et al., 2019).

## *Drosophila* models of myocardial contractility dysfunction

Like CHD, cardiomyopathies are genetically and phenotypically diverse (Arad et al., 2002; Richard et al., 2006; Ware et al., 2021). Among the many cardiomyopathy-associated genes is *Lamin A/C* (*LMNA*), one of the most sequenced human genes. It has hundreds of variants associated with multiple cardiomyopathies including those with pediatric onset (Heller et al., 2017; Kervella et al., 2022). Cardiomyopathy clinically manifests as systolic and diastolic dysfunction, arrhythmia, and increased risk of heart failure. These functional manifestations have been linked to structural issues of increased ventricular size, thickening of the ventricles (*i.e.*, hypertrophy), and stiffening of the ventricular walls (Lee et al., 2017; El Hadi et al., 2023). Multiple structural and functional readouts that have been established for the fly heart can capture these phenotypes. Brightfield microscopy of histological sections or micro computerized tomography, a 3D X-ray imaging technique, can be used to determine the thickness of the heart muscle wall (Migunova et al., 2021; Petersen et al., 2022). Whereas high-speed movies of semi intact *Drosophila* heart preparations (Ocorr et al., 2007), *in vivo* imaging of the heart in intact flies using high resolution optical coherence microscopy which yields imaging similar to ultrasound (Migunova et al., 2021), or optical coherence tomography which is similar to echocardiography in humans (Wolf et al., 2006), can be used to quantify muscle wall thickness and function, including diastolic diameter, end systolic diameter, and fractional shortening. Finally, the cardiac flow, a measure of contractile force, can be measured by a dye injection assay that times the flow from injection site to target site (Zhu et al., 2017a; 2017b), or with intravital imaging which enables life tracking of the heart wall, quantitation of the chamber diameter during contraction (systole) and relaxation (diastole), and fractional shortening, as well as estimates of cardiac output and stroke volume using segmentation algorithms (Klassen et al., 2017).

The above techniques to observe the heart in flies have been successfully used in fly models for diverse cardiomyopathies including modulators of the EGF receptor signaling pathway associated with dilated (Yu et al., 2010) or hypertrophic (Yu et al., 2013) cardiomyopathy; in which flies showed increased cardiac chamber size, and cardiac hypertrophy with reduced contraction, respectively. *Tropomyosin II null* (*TM2* ^*3*^) mutant flies showed cardiac arrhythmia reminiscent of the clinical observation in patients with Tropomyosin-associated dilated cardiomyopathy (Ma et al., 2010). Moreover, these techniques to study the fly heart have been applied to establish genetic causality for dilated cardiomyopathy using fly models deficient for *Phosphopantothenoylcysteine synthetase* (*PPCS*) and transgenic flies that caried *PPCS* with patient variants (Iuso et al., 2018). Affected flies showed reduced viability, increased heart rate, increased arrhythmia index, reduced systolic function, and increased heart wall shortening, reminiscent of the pathophysiology observed in the patients (Iuso et al., 2018). The methods to observe fly hearts have also been applied to establish causality for *Zinc phosphodiesterase ELAC protein 2* (*ELAC2*) genetic variants in a rare form of severe infantile cardiomyopathy (Migunova et al., 2021). Transgenic flies expressing patient mutations in the homologous fly gene (*RNaseZ*) displayed cardiac hypertrophy and reduced contraction mimicking the clinical pathology in patients (Migunova et al., 2021). In a final example, the techniques to study the heart in fly were used to establish causality for variants in *Myosin light chain 2 (MYL2)* associated with hypertrophic cardiomyopathy of infantile onset and characterized by mitral valve dysplasia, resulting in infant death (Manivannan et al., 2020). Silencing the fly homologous gene *Mlc2* in the fly heart resulted in developmental lethality and decreased fractional shortening similar to the patients that carried loss-of-function variants (Manivannan et al., 2020). These phenotypes could be rescued by expressing wildtype human *MYL2*, but not by *MYL2* patient variant cDNA (Manivannan et al., 2020). These studies exemplify both the relevance and the potential of using fly models to study myocardial contractility defects.

## *Drosophila* models of metabolic syndrome associated heart diseases

Nearly all metabolic pathways are shared between flies and humans (Bharucha, 2009), making fly a notable model system to study metabolic and diet-associated diseases, such as obesity (high-fat diet fly models) and diabetes (high-sugar diet fly models). Obesity caused by high-fat diet is a major contributor to diabetes and related cardiovascular complications. In patients, obesity-associated cardiomyopathy is marked by altered metabolism, ventricular hypertrophy and remodeling, diastolic and systolic dysfunction, abnormal conduction, atrial fibrillation (*i.e.*, arrhythmia), and ultimately heart failure (Piché et al., 2020). Flies fed a high-fat-diet mimic the features of metabolic syndrome in patients, including elevated lipid levels and altered insulin and glucose homeostasis (Birse et al., 2010). Moreover, the diet has detrimental effects on the fly heart, including reduced contractility, conduction blocks (*i.e.*, anterior and posterior heart beat at different rates), dysfunctional ostia (valves at the inflow tracts), and structural defects and disorganization of the myofibrils (Birse et al., 2010). Moreover, the cardiac dysfunction induced by high-fat-diet in *Drosophila* was shown to persist for two subsequent generations (Guida et al., 2019). This heredity was linked to lasting metabolic changes mediated by epigenetic modifications (Guida et al., 2019). Many gaps remain in our understanding of the pathomechanisms underlying obesity-induced cardiomyopathy and the inherited risks for future generations; *Drosophila* high-fat-diet models could provide a valuable contribution to this research.

Incentivized by the conserved metabolic pathways and the range of genetic tools available in fly, *Drosophila* models for type I and type II diabetes have been established. Glucose homeostasis is highly conserved and includes fly functional equivalents of mammalian insulin and glucagon (Liguori et al., 2021). Similar to patients, diabetic flies display signs of hyperglycemia, hypertrehalosemia, peripheral resistance to exogenous insulin, and accumulation of triglyceride (Na et al., 2013). Flies on a high-sucrose diet displayed increased cardiac arrhythmia, and increased diastole and systole (without fractional shortening) (Na et al., 2013) reminiscent of diabetes-associated dilated cardiomyopathy. The functional outcomes were accompanied by structural defects, including increased Pericardin deposition in fly heart tissue, a measure of cardiac fibrosis (Na et al., 2013) which is a common complication in patients with diabetes (Armstrong et al., 2017). These cardiac symptoms in fly were more severe at higher dietary sugar content and with age/prolonged exposure, ultimately resulting in a shortened lifespan (Na et al., 2013). Besides genetic factors, environmental factors greatly contribute to the risk of developing diabetes. *Drosophila* can be a powerful model in studying these gene-environment interactions. For example, maternal diabetes-induced fetal hyperglycemia is associated with a five-fold increased risk for CHD. Combining an assay for transposase-accessible chromatin with high-throughput sequencing (ATAC-seq) of a high-glucose *in vitro* model, and maternal diabetic mouse and *Drosophila* models, revealed a conserved interaction between Notch1 signaling (gene) and high-glucose (environment); controlled by a Jarid2 (repressor of Notch signaling)-mediated epigenetic mechanism (Basu et al., 2017). This study demonstrates that fly diabetic models can be valuable tools in unraveling the gene-environment interactions that underly diabetes-associated cardiomyopathy.

## *Drosophila* models of cardiac aging

Aging is associated with increased risk for cardiovascular disease, including increased prevalence of atrial fibrillation (*i.e.*, arrhythmia) and decreased diastolic function of the left ventricle, even in the absence of major cardiovascular risk factors (Dai et al., 2012; Gude et al., 2018; Go et al., 2023). Similarly, in *Drosophila*, aging leads to decreased cardiac output (Klassen et al., 2017) and increased cardiac arrhythmia (Ocorr et al., 2007; Taghli-Lamallem et al., 2008). Due to the flies’ relatively short lifespan, with a median lifetime of 63 days (Taghli-Lamallem et al., 2008), aging-related studies of cardiac morphology and function can be accomplished within practicable timeframes. Electrophysiology can be used to measure cardiac action potentials, like electrocardiography in humans (Johnson et al., 2001; Papaefthmiou and Theophilidis, 2001; Lalevée et al., 2006). Aging flies displayed a reduced resting heart rate and increased cardiac arrhythmia events, as well as cardiovascular stress-induced maximal heart rate and increased heart failure (Paternostro et al., 2001; Wessells et al., 2004). These findings are similar to the cardiac functional decline observed in the aging human heart (Dai et al., 2012; Gude et al., 2018; Go et al., 2023).

Fly models of aging have also been used to study the molecular-genetic pathways that underly age-related cardiac pathophysiology. The insulin and mTOR pathways play key regulatory roles in aging (Kim, 2007; Papadopoli et al., 2019), and their disruption leads to similar defects of the heart in aging flies (Wessells et al., 2004; Luong et al., 2006). A fly study identified several effectors that act at the interchange of insulin and mTOR signaling, and demonstrated their importance during age-related cardiac decline (Wessells et al., 2009). Another study of the aging fly heart found that cardiac decline and arrhythmias were accompanied by reduced expression of *KCNQ* (fly homolog of mammalian *KCNQ1*-encoded voltage-gated potassium channel alpha subunits) (Ocorr et al., 2007). Moreover, hearts of young flies deficient for *KCNQ* displayed prolonged contractions and fibrillation, reminiscent of the cardiac arrhythmic phenotypes in patients with mutations in *KCNQ1* (torsade de pointes/congenital long QT syndrome) (Ocorr et al., 2007). A comparative study of age-related cardiac transcriptomic changes identified pathways involved in remodeling of the extra-cellular matrix, mitochondrial metabolism, protein handling, and contractile functions, that were conserved between *Drosophila* and rodents (Cannon et al., 2017). Besides, like individual aging rodent hearts, the gene expression changes between individual aging fly hearts showed little overlap. The findings suggest that different transcriptional paths can lead to similar age-related cardiac decline (Cannon et al., 2017). Taken together, these studies demonstrate the proficiency of the *Drosophila* system to model the physiology underlying the aging heart in the absence of major cardiovascular risks.

## Discussion

Decades of research contributions from *Drosophila* models have taught us much about the developing and aging heart in health and disease. However, several challenges remain, which when overcome would greatly expand the opportunities to use fly models in studies of cardiac disease. Whereas single-cell RNA sequencing technology has provided detailed transcriptomic profiles of the developing heart in humans (Asp et al., 2019; Cui et al., 2019; Litvinukova et al., 2020; Tucker et al., 2020) and several model systems, including mouse (DeLaughter et al., 2016; Li et al., 2016; Li et al., 2019b.; Li et al., 2019a.; Gladka et al., 2018; Hu et al., 2018; Jia et al., 2018; Lescroart et al., 2018; Skelly et al., 2018; Farbehi et al., 2019; Goodyer et al., 2019), zebrafish (*Danio rerio*) (Burkhard and Bakkers, 2018; Yuan et al., 2018; Honkoop et al., 2019; Weinberger et al., 2020), and sea squirt (*Ciona robusta*) (Wang et al., 2019); for *Drosophila* single-cell RNA sequencing data is only available for the adult heart (Li et al., 2022a). Knowing the cell types that make up the fly heart at crucial developmental stages would facilitate determining the extent of evolutionary conservation of heart development and its underlying molecular pathways. In addition, these data would enable more direct comparisons between different cardiac cell types in flies and human. This knowledge could be used to better assign disease subtypes, especially those with genotypic and phenotypic overlap; for example, by targeting RNAi and/or human cDNA carrying patient variants to the fly equivalent of the cells affected in patients. Together, the better understanding would aid translation of the findings in fly to applications in patients.

It is worth noting that to accommodate its open circulatory system, the fly carries additional pulsatile organs, known as antennal/frontal accessory pulsatile organ and wing hearts, that ensure circulation of hemolymph (fly blood) throughout its antennae and wings, respectively (Tögel et al., 2013; Hillyer and Pass, 2020; Kay et al., 2021). The muscle cells that make up the wing hearts originate from a select group of pericardial progenitors, marked by the expression of Even skipped (Eve) and early loss of Tinman (Tögel et al., 2008). Pathological conditions that affect the equivalent cell in the human heart—single-cell RNA sequencing data of the fly, mammalian, and human hearts could shed light on this—could potentially benefit from studying the fly wing hearts specifically.

Arrhythmia can present as either a primary or a secondary cause. For example, as a complication in diverse CHD and cardiomyopathies, as well as in the aging heart in the absence of major cardiovascular risk factors. A clear pathophysiological understanding is crucial to distinguish between arrhythmia as a primary or secondary feature. In humans, the heart’s rhythm is regulated by the sinoatrial node (SA node), the atrioventricular node (AV node), the bundle of His, and Purkinje fibers which make up the cardiac conduction system (Chloe Li et al., 2022). The SA node acts as the biological pacemaker, its excitation signals the start of a heartbeat (Hanna et al., 2021). The SA node is controlled by the vagus nerve which innervates both the heart muscle cells and the conduction system (Capilupi et al., 2020). However, the exact neural circuitry and the molecular mechanisms that coordinate the parasympathetic (relaxes heart rate) and sympathetic (increases heart rate) actions of the vagus nerve are not fully understood. Likewise, the *Drosophila* heart is innervated by peripheral neurons (Dulcis and Levine, 2005) (Figure 1). Although, many questions remain regards the fly heart conduction system. A study into myotonic dystrophy type 1 (DM1) demonstrates the potential of *Drosophila* to model human cardiac conduction defects. The study simulated the DM1-associated misbalance between two RNA binding factors, Muscleblind like splicing regulator (MBNL1) and CUGBP Elav-like family member 1 (CELF1), in the fly heart; this led to dysregulated calcium signaling genes including *straightjacket* (*stj*)/*α2δ3*, which encodes a voltage-gated calcium channel subunit (Auxerre-Plantié et al., 2019). In the flies, dysregulated *stj* resulted in an asynchronous heartbeat, indicative of abnormal conduction. Moreover, the study showed altered expression of *α2δ3* in heart tissue from patients with DM1-associated conduction defects (Auxerre-Plantié et al., 2019). Better understanding the similarities and differences between the human and fly cardiac conduction systems would aid the development of additional fly models for arrhythmia. A variety of imaging tools to assay the heart cycle and rhythm (Wolf et al., 2006; Ocorr et al., 2007; Zhu et al., 2017a; Klassen et al., 2017; Migunova et al., 2021) and electrophysiology to measure the energy fluxes (Johnson et al., 2001; Papaefthmiou and Theophilidis, 2001; Lalevée et al., 2006) are already available and will aid this research direction.

In addition to accommodating studies of gene-environment interactions, like fly models for diabetes, *Drosophila* provides an excellent opportunity to study polygenic causation, which has likewise been difficult to study using conventional animal models. However, Genomic studies have found many patients with CHD or cardiomyopathy that likely have a polygenic cause (Zaidi et al., 2013; Homsy et al., 2015; Jin et al., 2017; Ware et al., 2021). For these studies too, the fly could be immensely valuable. In fact, several polygenic fly models for cardiac disease have been generated (Qian and Bodmer, 2012), thus demonstrating feasibility. The numerous readily available transgenic fly lines, unmatched genetic tools, and rapid crosses with large progenies make for relatively straightforward development of polygenic fly models that carry the genetic variant combinations identified in patients. Moreover, the compact *Drosophila* genome carries little redundancy which facilitates data interpretation. These studies could reveal new disease mechanisms relevant to the human heart.

The flies’ established track record combined with the latest technology and assays for fly cardiac function, and the many new avenues about to be explored make *Drosophila* a very exciting model system to study a wide variety of aspects of human cardiac diseases.

## Data Availability

The original contributions presented in the study are included in the article/supplementary material, further inquiries can be directed to the corresponding author.
